# Association of Yogurt Consumption with Nutrient Intakes, Nutrient Adequacy, and Diet Quality in American Children and Adults

**DOI:** 10.3390/nu12113435

**Published:** 2020-11-09

**Authors:** Christopher J. Cifelli, Sanjiv Agarwal, Victor L. Fulgoni

**Affiliations:** 1 National Dairy Council, 10255 West Higgins Road, Suite 900, Rosemont, IL 60018-5616, USA; 2 NutriScience LLC, East Norriton, PA 19403, USA; agarwal47@yahoo.com; 3 Nutrition Impact, LLC, Battle Creek, MI 49014, USA; VIC3RD@aol.com

**Keywords:** national health and nutrition examination survey, NHANES, healthy eating index, HEI, BMI, overweight, obese

## Abstract

The popularity of yogurt has increased among consumers due to its perceived health benefits. This study examined the cross-sectional association between yogurt consumption and nutrient intake/adequacy, dietary quality, and body weight in children and adults. National Health and Nutrition Examination Survey 2001–2016 data (*n* = 65,799) were used and yogurt consumers were defined as those having any amount of yogurt during in-person 24-h diet recall. Usual intakes of nutrients were determined using the National Cancer Institute method and diet quality was calculated using the Healthy Eating Index-2015 (HEI-2015) scores after adjusting data for demographic and lifestyle factors. The data show that approximately 6.4% children and 5.5% adults consume yogurt, with a mean intake of yogurt of 150 ± 3 and 182 ± 3 g/d, respectively. Yogurt consumers had higher diet quality (10.3% and 15.2% higher HEI-2015 scores for children and adults, respectively); higher intakes of fiber, calcium, magnesium, potassium, and vitamin D; and higher percent of the population meeting recommended intakes for calcium, magnesium, and potassium than non-consumers. Consumption of yogurt was also associated with lower body weight, body mass index (BMI), and 23% showed a lower risk of being overweight/obese among adults only. In conclusion, yogurt consumption was associated with higher nutrient intake, nutrient adequacy, and diet quality in both children and adults.

## 1. Introduction

Fermented foods have played an important role in human health for centuries because of their enhanced preservation and functional properties [1]. The popularity of fermented foods has steadily increased among consumers because of their link to improved health. Yogurt is a semisolid fermented milk product produced by lactic acid–producing bacteria *Lactobacillus bulgaricus* and *Streptococcus thermophilus* [2]. Similar to other fermented foods, yogurt production has increased by over 4% between 1995 and 2019 [3] and, correspondingly, yogurt intake has steadily increased in the past decade [4]. Yogurt has higher amounts of protein, vitamin B_2_, vitamin B_12_, calcium, magnesium, potassium, and zinc than milk [5]. Yogurt may provide additional health benefits beyond nutrient provision because it contains unique bioactive compounds and live and active cultures [6].

An accumulating body of scientific evidence suggests that yogurt and fermented dairy consumption may be associated with improved cardiometabolic health [7,8,9]. Meta-analyses have shown that yogurt intake is linked with lower risk of type 2 diabetes [10,11], cardiovascular disease [12], metabolic syndrome [13], and with lower risk of cardiovascular- and all-cause mortality [14]. Higher yogurt consumption was associated with a 16% lower risk of high blood pressure in a long-term cohort study [15] and each additional serving of yogurt was associated with a 6% lower risk of incident hypertension in a Framingham Heart Study cohort [16]. Yogurt consumption has also been shown to be associated with a lower body mass index (BMI), lower body weight/weight gain, smaller waist circumference (WC), and lower body fat [17,18,19,20]. It has been hypothesized that the formation of unique bioactive compounds during fermentation, the presence of live and active cultures in fermented dairy, and conjugated linoleic acid of certain whole-milk yogurts are responsible for the beneficial associations between yogurt and cardiometabolic health [7,8].

The relationship between yogurt consumption and nutrient adequacy and markers of cardiometabolic health has been previously investigated. Hobbs et al. [21] reported higher diet quality, nutrient intakes, and nutrient adequacy among British children consuming >60 g/d yogurt. Vatanparast et al. [22] also reported a higher nutrient intake and diet quality among Canadian children and adult yogurt consumers. Similarly, some studies have examined the possible links between yogurt consumption and nutrient adequacy and body weight in the US population. Wang et al. [23] reported that yogurt consumption was associated with better diet quality, higher nutrient intakes, and healthier metabolic profiles in adults from the Framingham Heart Study. Data from the National Health and Nutrition Examination Survey (NHANES) showed that yogurt was associated with higher nutrient intake, diet quality scores, and better metabolic profiles in children [24,25]. However, neither NHANES analysis examined the impact of yogurt consumption in adults.

Given the increase in popularity and consumption of yogurt in recent years, there is a need to more fully understand the impact of yogurt on health outcomes in both adults and children. Accordingly, the aim of the current study was to investigate the association between yogurt consumption, nutrient adequacy, diet quality, and body weight in American children and adults using the NHANES data set. We hypothesized that yogurt consumption would be associated with greater nutrient intakes/adequacy, improved diet quality, and lower body weight as compared to non-consumers.

## 2. Materials and Methods

### 2.1. Subjects

Food and nutrient data obtained from the dietary component of What We Eat In America (WWEIA) of NHANES 2001–2016 were used to assess yogurt intake [26]. NHANES is a continuous survey of a nationally representative sample of the non-institutionalized US population conducted by the National Center for Health Statistics (NCHS). The present analysis combined 8 NHANES datasets (NHANES 2001–2002, 2003–2004, 2005–2006, 2007–2008, 2009–2010, 2011–2012, 2013–2014, and 2015–2016). The combined sample included 65,799 participants aged 2 years and over, excluding pregnant females (*n* = 1356), lactating females (*n* = 338), and those with incomplete or unreliable 24-h recall data (8622). All participants provided written informed consent and the Research Ethics Review Board at the NCHS approved the survey protocol. The federal NHANES database, which is publicly available at [27], is exempt from approvals by Institutional Review Boards.

### 2.2. Estimation of Energy and Nutrients Intakes

Dietary intake data with reliable 24-h recall dietary interviews using the United States Department of Agriculture’s (USDA) automated multiple-pass method were used [28]. The 24-h recall data for each participant in these surveys includes a description of the individual foods and beverages consumed on the previous day (midnight to midnight) and the amount by weight. Complete descriptions of the dietary interview methods for NHANES are provided elsewhere [26]. Yogurt was defined as g of food in “USDA food subgroup 18” and includes both plain and flavored, Greek and regular, and all fat varieties of yogurt. [29]. Yogurt consumers were defined as those consuming any amount of yogurt during the first (in-person) 24-h recall. Participants were dichotomized into consumers and non-consumers of yogurt. The second dietary recall was used to assess usual nutrient intakes. Energy and nutrients for each food and beverage consumed were determined using the NHANES cycle specific the USDA Food and Nutrient Database for Dietary Studies (FNDDS) [29]. In our analysis, intake of calcium, iron, magnesium, potassium, thiamin, folate, and vitamins A, B_6_, B_12_, C, and D was analyzed as these are defined as “under consumed nutrients” by the Dietary Guidelines for Americans 2015–2020 (DGA) [30].

### 2.3. Estimation of Diet Quality and Intake of Food Groups

Diet quality scores were determined using the USDA Healthy Eating Index-2015 (HEI-2015) [31]. The HEI-2015 contains 13 subcomponents, each reflecting the DGA’s recommendations. HEI-2015 scores were estimated using day 1 dietary intake data. Dietary intake was expressed per 1000 kcal for all components except for fatty acid ratios (expressed as a ratio of unsaturated to saturated fatty acids), saturated fat (expressed as % energy), and added sugars (expressed as % energy). Total vegetables; greens and beans; total fruit, whole fruit; total protein; and seafoods and plant proteins were scored proportionally from 0 to 5 points and all other components (i.e., whole grains; dairy; fatty acids; sodium; refined grains; saturated fat; and added sugars) were scored proportionally from 0 to 10 points. The maximum possible score was 100 [31].

The USDA Food Patterns Equivalents Database (FPED) [32] was used to calculate an intake of MyPlate [33] servings (MyPyramid Equivalents Database was used for 2001–2004 data). The number of MyPlate servings was aggregated over all foods consumed during the 24-h recall to calculate the MyPlate food group intakes per day.

### 2.4. Estimation of Anthropometric Measures

Body weight, waist circumference, body mass index (BMI) for adults, and BMI z-score for children were calculated using NHANES standard protocols and using the Statistical Analysis Software program for CDC and Prevention’s Growth Charts [26,34]. In adults overweight or obese the BMI was defined as ≥25 kg/m^2^ and the elevated waist circumference was defined as >102 cm for males and >88 cm for females [35]. Children with BMI z-scores between the 85th and <95th percentile were considered overweight, and those with BMI z-scores in the ≥95th percentile were considered obese [34].

### 2.5. Statistical Analysis

All analyses were performed using SAS 9.4 (SAS Institute, Cary, NC, USA) software. The data were adjusted for the complex sampling design of NHANES, using appropriate survey weights, strata, and primary sampling units. Day one dietary/examination weights were used in all intake analysis and Mobile Examination Center weights were used for anthropometric variables.

Least square means (LSM) and standard errors (SE) were generated for energy and nutrient intake, food group intake, diet quality, and anthropometric variables in yogurt consumers and non-consumers via regression analyses. Analyses were adjusted for age, gender, ethnicity, poverty income ratio, physical activity level, current smoking status, and alcohol (only for those 19 years and older), and kcal (except for energy and HEI-2015). Usual intakes of nutrients were determined using the National Cancer Institute (NCI) method [36] and the NCI macros (Mixtran and Distrib) were used to generate parameter effects after covariate adjustments and to estimate the distribution of usual intake (UI). The one-part NCI model was used for nutrients since these substances are consumed on most days by most subjects. Covariates for usual intake estimation included day of the week of the 24-h recall [coded as weekend (Friday–Sunday) or weekday (Monday–Thursday)] and sequence of dietary recall (first or second), and variance estimates were obtained using the two days of intake with one-day sampling weights. The percentage of the population below the Estimated Average Requirement (EAR) or above Adequate Intake (AI) of nutrients was assessed using the cut-point method (except for iron where the probability method was used) and Z-statistic was used to assess differences between non-consumers and yogurt consumers. Logistic regression was utilized to assess the association of yogurt consumption with risk of overweight/obesity in children and adults. *p* < 0.01 was considered statistically significant.

## 3. Results

### 3.1. Yogurt Intake

Approximately 6.4% of children and 5.5% of adults consume yogurt. Yogurt consumers were 3.0 and 2.6 years younger than non-consumers in children and adults, respectively. While in children a similar percentage of males/females consume yogurt, a smaller percentage of adult males consume yogurt. A greater percentage of yogurt consumers were non-Hispanic White (and a lower percentage yogurt consumer were non-Hispanic Blacks) as compared to non-consumers in both children and adults. A greater percentage of yogurt consumers had a household income >1.85 the poverty level and a lower percentage yogurt consumer had a household income <1.35 the poverty level as compared to non-consumers in both children and adults (Supplementary Table S1). The mean intake of yogurt (on day 1 of recall) among consumers was 150 ± 3 g/d (95th percentile 307 g/d) and 182 ± 3 g/d (95th percentile 337 g/d) among children and adults, respectively. Median intake was 122 g/d in children and 169 g/d in adults. Mean per capita intake of yogurt (on day 1 of recall) was 11.6 ± 0.5 and 12.9 ± 0.5 g/day among children and adults, respectively, and has significantly increased (β = 0.59 g/cycle, *p* = 0.0164 for children; β = 1.46, g/cycle *p* < 0.0001 for adults) over the last eight NHANES cycles (2001–2016).

Yogurt provided on average 7.7% of energy, 18.5% of calcium, 10.5% of vitamin D, 12.3% of potassium, 14.3% of vitamin B_12_, 10.4% of protein, 16.9% of total sugars, and 17.9% of added sugars in children (Table 1). Similar results were observed in adults where, on average, yogurt provided 7.6% of energy, 21.7% of calcium, 15.5% of vitamin D, 11.2% of potassium, 15.6% of vitamin B_12_, 11.0% of protein, 17.5% of total sugars, and 16.3% of added sugars (Table 1).

### 3.2. Comparison Between Yogurt Consumers vs. Non-Consumers on Energy and Nutrient Intakes

There were significant differences in nutrient intake between yogurt consumers and non-consumers (Table 2). In children, yogurt consumers had higher intakes of carbohydrate (2.3%), dietary fiber (4.5%), total sugars (9.3%), protein (4.9%), calcium (21.4%), magnesium (10.0%), potassium (11.1%), vitamin B_12_ (7.1%), and vitamin D (7.6%), as well as lower intakes of total fat (−5.2%), and sodium (−5.7%) than non-consumers (*p* < 0.01 for all). Similarly, adult yogurt consumers had higher intakes of energy (4.9%), carbohydrate (4.7%), dietary fiber (15.9%), total sugars (9.6%), protein (7.0%), calcium (30.3%), magnesium (17.1%), potassium (15.3%), vitamin A (11.5%), folate (9.8%), vitamin B_6_ (8.9%), vitamin B_12_ (13.5%), vitamin C (19.2%), and vitamin D (13.7%), as well as lower intakes of added sugars (−6.7%), total fat (−8.5%), cholesterol (−12.5%). and sodium (−6.3%) than non-consumers (*p* < 0.01 for all). The intake of other nutrients was not significantly different among yogurt consumers and non-consumers (Table 2).

A lower proportion of yogurt consumers compared to non-consumers (*p* < 0.01) were below the EAR for six nutrients for children and four nutrients for adults out of the 10 nutrients examined. Yogurt consumers had at least a 10 percentage unit difference in the population below the EAR for calcium (−33.2% and −27.6% for children and adults, respectively), magnesium (−22.8% and −25.4% for children and adults, respectively), and vitamin A (−18.6% for both children and adults) when compared to non-consumers. The percentage above the AI for fiber among adults and for potassium for both children and adults were higher (*p* < 0.0001) among yogurt consumers compared to non-consumers (Table 3).

### 3.3. Link Between Yogurt Intake and Diet Quality

Intake of yogurt was associated with 10.3% and 15.1% higher HEI-2015 scores (p < 0.01) in children and adults, respectively (Table 4). The HEI-2015 subcomponent scores for ‘greens and beans,’ ‘total fruit,’ ‘whole fruit,’ ‘whole grain,’ ‘dairy,’ ‘seafood and plant protein,’ ‘sodium,’ ‘refined grains,’ and ‘saturated fat’ were higher (p < 0.01) in consumers compared to non-consumers for both children and adults. Additionally, adult yogurt consumers had a higher score for ‘added sugar’ and a lower score for ‘fatty acids ratios’ (*p* < 0.01 for all).

**Figure 1:**
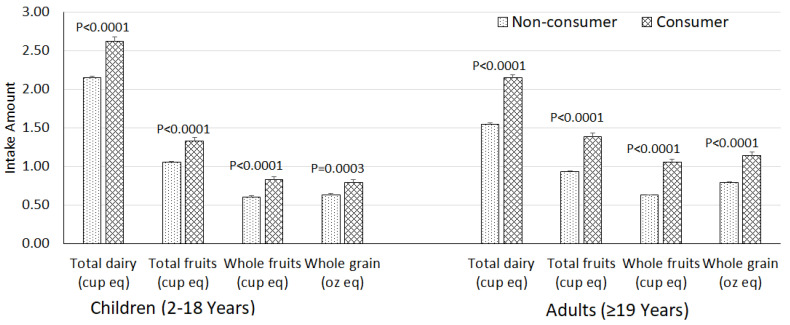
Covariate-adjusted select food groups intakes in children and adult yogurt consumers and non-consumers (NHANES 2001–2016, gender combined data). Values are least square means ± standard error of means, adjusted for age, gender, ethnicity, poverty income ratio, physical activity level, current smoking status, and alcohol (only for ≥19 years), and kcal. *p* values are for difference between consumers and non-consumers.

Intake of yogurt was also linked with significant differences (*p* < 0.01) in specific MyPlate food groups [33]. Yogurt consumers compared to non-consumers had higher intakes of total dairy (+21.9% in children; +38.7% in adults), total fruit (+26.7% in children; +49.5% in adults), whole fruit (+36.7% in children; +66.7% in adults), and whole grain (+25.4% in children; +44.3% in adults) (Figure 1).

### 3.4. Association Between Yogurt Intake and Anthropometric Measures

Anthropometric data are presented in Table 5. Yogurt consumption was inversely associated with BMI (−0.9 kg/m^2^, *p* < 0.0001), body weight (−2.1 kg, *p* = 0.0004), and waist circumference (−2.3 cm, *p* < 0.0001) among adults. Furthermore, among adults, yogurt consumers had a 23% lower risk of being overweight or obese (OR: 0.77; 99% CI: 0.65, 0.91; *p* = 0.0001) and a 29% lower risk of having an elevated waist circumference (OR: 0.71; 99% CI: 0.60, 0.85; *p* < 0.0001) compared to non-consumers. There were no differences in anthropometric measures between yogurt consumers and non-consumers in children.

## 4. Discussion

This is the first report to investigate the association between yogurt consumption and nutrient intakes, nutrient adequacy, diet quality, and weight status in a nationally representative population of American children and adults. Combining data from eight cycles of NHANES, the present analysis showed that yogurt consumption was associated with higher nutrient intakes and better nutrient adequacy, as well as a higher diet quality score compared with non-consumers for both children and adults. Furthermore, weight-related outcomes were better in adult yogurt consumers when compared to non-consumers.

Approximately 6% of Americans consumed yogurt on day 1 of the NHANES 24-h dietary recall. The prevalence of yogurt consumption in American children and adults in the present analysis is less than recent estimates in British and Canadian population groups [21,22]. Using data from the National Diet and Nutrition Survey, Hobbs et al. [21] reported that about 62% of children aged 4–10 years and 31% of children aged 11–18 years were yogurt consumers. Similarly, Vatanparast et al. [22] analyzed data from Canadian Community Health Survey 2015 and found 20% Canadians consumed yogurt on a given day. The prevalence of yogurt consumption observed in this study is close to those by Keast et al. [25], who estimated that 8.5% of children and adolescents age 8–11 years were yogurt based on data from NHANES 2005–2008. Other estimates indicated that 33% children age 2–18 years (NHANES 2003–2006), and 41% men and 64% women (Framingham Heart Study Cohorts 1998–2001, 2002–2005) consumed yogurt at least once per week [23,24]. While the prevalence observed in our analysis appears low, on a population basis this represents about 12 million individuals. The mean intake of yogurt in this study are higher than the 135 g/d intake estimate for the Canadian population [22] and a 108 g/d intake tertile II estimate for 8–11-year-old British children [21]. In this analysis, the mean intake of yogurt among consumers was 5.3 fluid oz/d (0.66 cups or 162 g) for children and 6.4 fluid oz/d (0.8 cups or 196 g) for adults, which represents approximately 0.9 servings per day for children and 1.1 servings per day for adults. Thus, regular yogurt consumption may significantly contribute to meeting the DGA recommendations for dairy foods.

Yogurt consumers had significantly higher intakes of fiber, calcium, magnesium, potassium, vitamin A, vitamin B_6_, vitamin B_12_, vitamin C, and vitamin D than non-consumers. Along those lines, yogurt consumers also had a higher nutrient adequacy for fiber, calcium, magnesium, potassium, vitamin A, and vitamin B_12_ than non-consumers. Many of these nutrients are currently under-consumed by Americans and have been identified as “shortfall nutrients” by the DGA [30]. Additionally, the DGA has classified dietary fiber, calcium, potassium, and vitamin D as “nutrients of public health concern” because their current intakes are low enough to impact one’s health [30]. Similar observations were also reported in earlier cross-sectional studies from both US and international cohorts [21,22,23,24,25]. Since yogurt is a good source of several of the above nutrients [37,38,39,40,41], yogurt consumption is naturally expected to lead to more nutrient dense diets and greater adequacy for nutrients. Finally, children and adult consumers of yogurt compared to non-consumers had 176 and 223 mg/d lower intakes of sodium respectively. High sodium intake has been linked to elevated blood pressure and therefore reducing dietary sodium is an important target for public health improvement [30].

As yogurt is not a good source of fiber and vitamin C, the results suggest that yogurt consumers are eating higher amounts of other healthy foods as well. Indeed, this was reflected in the fact that both children and adult yogurt consumers had better diet quality scores than non-consumers. Diet quality was assessed by HEI-2015 [31] in the present analysis. HEI is a validated measure of diet quality and is indicative of compliance/adherence of a person’s diet to the eating pattern recommended by the DGA [30]. HEI is commonly used to evaluate diets in population groups [42], food environments [43], to assess changes in the diet quality over time [44], and to validate other nutrition research tools and indexes [45]. It has also been used in recent research to understand relationships between nutrients/foods/dietary patterns and health-related outcomes [46,47,48,49]. In the present analysis, HEI-2015 total scores of yogurt consumers were significantly higher for children and adults than their respective non-consumers, indicating a higher compliance to nutritional guidelines. These results are consistent with earlier cross-sectional analyses, which found that yogurt consumers have a significantly higher diet quality than non-consumers [21,22,23,24,25]. HEI-2015 has 13 subcomponents (nine for adequacy and four for moderation) [31] and the scores for 9 and 10 subcomponents were also significantly higher for children and adult consumers. It is interesting to note that not only were the HEI-2015 subcomponent scores related to dairy, fruits, vegetables, and whole grain higher, the actual intakes of these food groups were also significantly higher in yogurt consumers than non-consumers. This is important because current intakes of these food groups are lower than the recommended amounts in the USDA’s Healthy US-Style Eating Pattern [30]. Indeed, the majority of the US population currently does not meet the daily intake recommendations for fruits (nearly 80%), vegetables (nearly 90%), whole grain (nearly 100%), and dairy (nearly 80%) [37].

In the current study, adult yogurt consumers had a lower weight, BMI, and waist circumference than non-consumers and yogurt consumption was significantly associated with lower odds of being overweight or obese and having an elevated waist circumference. This is an interesting finding since adult yogurt consumers also had about a 5% higher calorie intake. One potential explanation for this observation is the presence of live and active cultures in yogurt. Yogurt contains the starter cultures *S. thermophilus* and the *L. delbrueckii* subspecies *bulgaricus* which are thought to contribute to heath. Other *Bifidobacterium* and *Lactobacillus* strains are sometimes added as probiotics, which are live microorganisms that when consumed in adequate amounts confer a health benefit in the host [50]. It has been hypothesized that the either the starter cultures, the probiotics, or the combination can beneficially impact the gut microbiota composition and function [6,51]. Thus, the observed link between yogurt consumption and better body composition could be driven by changes in the microbiota that are impacting energy metabolism. Since the present analysis did not distinguish between those yogurts that contain just the starter cultures and those with added probiotics, nor did it compare yogurts with live cultures to pasteurized products, additional work is needed to further elucidate the role of fermentation-associated microbes on weight and body composition. A healthier dietary pattern (higher intakes of fruits and vegetables, whole grain, and dairy) as observed in our analysis for yogurt consumers, along with potentially other lifestyle differences (e.g., more physical activity among yogurt consumers), could help explain the current findings of a lower body weight, BMI, and waist circumference and lowered risk for obesity. The results of this study are congruent with those from other cross-section studies that have shown that yogurt consumption was associated with a lower BMI, lower body weight/weight gain, and smaller waist circumference [21,22,25]. This finding may have important health and economic implications as more than one third of US adults are obese [52], and obesity is associated with several health risks [53] with annual medical costs amounting to $147 billion [54].

The regular consumption of added sugars from certain products has been associated with an increased risk of obesity [55,56,57]. The DGA recommends limiting intake of added sugars to less than 10% of total daily calories as part of a healthy diet [30]. More recently, the 2020 Dietary Guidelines Advisory Committee recommended that the intake of added sugars be lowered to 6% of total calories given their impact on health [58]. Flavored yogurts have the same nutrition profile as plain yogurts. The data presented herein showed that the intake of added sugars was nearly identical between non-consumers and consumers in both children and adults (Table 2), indicating that yogurt consumption was not linked to higher intakes of added sugars in this cohort. This study examined the short-term impact of yogurt intake on nutrient adequacy and body composition and did not distinguish between flavored and plain yogurts. Thus, additional work is needed to more fully understand the link between long term flavored yogurt intake and health.

The strengths of this study include the use of a large nationally representative sample achieved through combining several sets of NHANES data releases and the use of numerous covariates to adjust data to remove potential confounding. A major limitation of this study is the use of a cross-sectional study design, which cannot be used to determine cause and effect. The dietary intake data were self-reported recalls relying on memory and are potentially subject to reporting bias. Finally, while we accounted for a number of covariates in our statistical models, residual confounding cannot be ruled out.

## 5. Conclusions

In conclusion, the results showed that yogurt consumption was associated with higher nutrient intake, better nutrient adequacy, and better diet quality in both children and adults. Additionally, yogurt consumption was linked to a lower body weight and related parameters in adults. Long-term randomized controlled trials are needed to further examine the effects of yogurt consumption on body weight. Encouraging yogurt consumption may be an effective strategy for improving intakes and adequacy of certain nutrients and achieving a healthier dietary pattern.

## Figures and Tables

**Table 1:** Energy and nutrient intakes in children and adult yogurt consumers from all sources and from yogurt only (National Health and Nutrition Examination Survey (NHANES) 2001–2016, gender combined data).

Variable	Children (2–18 Years)		Adults (≥19 Years)	
	All sources	Yogurt only	All sources	Yogurt only
Energy (kcal)	1860 ± 25	144 ± 3	2114 ± 25	160 ± 2
Carbohydrate (g)	257 ± 3	24.6 ± 0.6	267 ± 4	25.7 ± 0.5
Dietary fiber (g)	13.5 ± 0.2	0.08 ± 0.01	19.8 ± 0.3	0.24 ± 0.02
Total sugars (g)	137 ± 2	23.2 ± 0.6	126 ± 2	22.0 ± 0.5
Added sugars (tsp eq)	17.6 ± 0.4	3.15 ± 0.09	16.3 ± 0.4	2.65 ± 0.09
Protein (g)	67.9 ± 1.2	7.07 ± 0.16	87.4 ± 1.2	9.59 ± 0.20
Total fat (g)	65.0 ± 1.3	2.15 ± 0.07	76.3 ± 1.2	2.25 ± 0.07
Cholesterol (mg)	198 ± 8	8.0 ± 0.2	249 ± 6	9.28 ± 0.22
Calcium (mg)	1227 ± 22	227 ± 5	1228 ± 17	267 ± 4
Iron (mg)	13.3 ± 0.3	0.12 ± 0.003	15.6 ± 0.3	0.16 ± 0.004
Magnesium (mg)	246 ± 3	22.4 ± 0.5	351 ± 5	26.9 ± 0.4
Potassium (mg)	2383 ± 35	294 ± 6	3133 ± 38	350 ± 5
Sodium (mg)	2719 ± 45	87.5 ± 1.8	3373 ± 47	103 ± 2
Vitamin A, RAE (µg)	639 ± 16	29.5 ± 2.0	749 ± 17	48.7 ± 2.2
Thiamin (mg)	1.47 ± 0.02	0.06 ± 0.001	1.69 ± 0.03	0.07 ± 0.001
Folate, DFE (µg)	521 ± 13	14.7 ± 0.4	600 ± 12	18.7 ± 0.4
Vitamin B_6_ (mg)	1.61 ± 0.03	0.06 ± 0.001	2.20 ± 0.05	0.08 ± 0.001
Vitamin B_12_ (µg)	5.12 ± 0.12	0.73 ± 0.01	5.88 ± 0.21	0.92 ± 0.01
Vitamin C (mg)	84.6 ± 3.0	1.45 ± 0.09	102 ± 3	2.43 ± 0.14
Vitamin D (µg)	6.57 ± 0.18	0.69 ± 0.03	5.41 ± 0.17	0.84 ± 0.03

Values are weighted means ± standard error of means. RAE, retinol activity equivalents; DFE, dietary folate equivalent.

**Table 2:** Covariate adjusted energy and nutrient intakes in children and adult yogurt consumers and non-consumers (NHANES 2001–2016, gender combined data).

Variables	Children (2–18 Years)			Adults (≥19 Years)		
	Non-Consumers (*n* = 22,355)	Consumers (*n* = 1557)	*p* Value for Difference	Non-Consumers (*n* = 34,711)	Consumers (*n* = 2024)	*p* Value for Difference
Energy (kcal)	1947 ± 9	1985 ± 26	0.1454	2142 ± 7	2247 ± 24	<0.0001
Carbohydrate (g)	262 ± 1	268 ± 1	0.0001	254 ± 1	266 ± 2	<0.0001
Dietary fiber (g)	13.3 ± 0.1	13.9 ± 0.2	0.0019	16.4 ± 0.1	19.0 ± 0.3	<0.0001
Total sugars (g)	129 ± 1	141 ± 2	<0.0001	115 ± 1	126 ± 1	<0.0001
Added sugars (tsp eq)	19.6 ± 0.1	19.3 ± 0.3	0.3875	18.0 ± 0.1	16.8 ± 0.3	0.0003
Protein (g)	68.8 ± 0.2	72.2 ± 0.7	<0.0001	81.4 ± 0.2	87.1 ± 0.9	<0.0001
Total fat (g)	73.1 ± 0.2	69.3 ± 0.6	<0.0001	81.5 ± 0.2	74.6 ± 0.7	<0.0001
Cholesterol (mg)	221 ± 2	214 ± 7	0.3622	288 ± 2	252 ± 6	<0.0001
Calcium (mg)	1001 ± 6	1215 ± 17	<0.0001	913 ± 4	1190 ± 13	<0.0001
Iron (mg)	14.3 ± 0.1	14.0 ± 0.2	0.2915	15.1 ± 0.1	15.2 ± 0.2	0.4588
Magnesium (mg)	230 ± 1	253 ± 2	<0.0001	293 ± 1	343 ± 3	<0.0001
Potassium (mg)	2184 ± 10	2427 ± 26	<0.0001	2666 ± 9	3074 ± 28	<0.0001
Sodium (mg)	3113 ± 12	2937 ± 30	<0.0001	3561 ± 9	3338 ± 30	<0.0001
Vitamin A, RAE (µg)	586 ± 5	623 ± 15	0.0202	626 ± 8	698 ± 14	<0.0001
Thiamin (mg)	1.55 ± 0.01	1.53 ± 0.02	0.3809	1.62 ± 0.01	1.65 ± 0.02	0.1538
Folate, DFE (µg)	522 ± 4	541 ± 12	0.1368	531 ± 3	583 ± 10	<0.0001
Vitamin B_6_ (mg)	1.70 ± 0.01	1.71 ± 0.03	0.8478	2.02 ± 0.01	2.20 ± 0.04	<0.0001
Vitamin B_12_ (µg)	4.93 ± 0.04	5.28 ± 0.10	0.0004	5.20 ± 0.05	5.90 ± 0.21	0.0016
Vitamin C (mg)	79.1 ± 1.0	85.4 ± 2.8	0.0271	83.7 ± 0.9	99.8 ± 3.1	<0.0001
Vitamin D (µg)	5.77 ± 0.05	6.21 ± 0.17	0.0092	4.60 ± 0.05	5.23 ± 0.16	0.0003

Values are least square means ± standard error of means, adjusted for age, gender, ethnicity, poverty income ratio, physical activity level, current smoking status, and alcohol (only for ≥19 years), and kcal (except for energy). *p* values are for difference between consumers and non-consumers. RAE, retinol activity equivalents; DFE, dietary folate equivalent.

**Table 3:** Nutrient adequacy in children and adult yogurt consumers and non-consumers (NHANES 2001–2016, gender combined data).

Variables	Children (2–18 Years)			Adults (≥19 Years)		
	Non-Consumers (*n* = 24,322)	Consumers (*n* = 1676)	*p* Value for Difference	Non-Consumers (*n* = 37,598)	Consumers (*n* = 2200)	*p* Value for Difference
	% population below Estimated Average Requirement (EAR)					
Calcium (mg)	48.6 ± 0.7	15.4 ± 1.9	<0.0001	48.3 ± 0.6	20.7 ± 1.7	<0.0001
Iron (mg)	2.18 ± 0.13	1.57 ± 0.47	0.0770	5.01 ± 0.12	5.51 ± 0.61	0.3147
Magnesium (mg)	37.0 ± 0.6	14.2 ± 1.0	<0.0001	58.0 ± 0.7	32.6 ± 1.5	<0.0001
Vitamin A, RAE (µg)	26.2 ± 0.9	7.58 ± 1.60	<0.0001	48.1 ± 0.8	29.5 ± 2.1	0.0004
Thiamin (mg)	1.34 ± 0.20	0.56 ± 0.27	0.0045	7.04 ± 0.41	5.47 ± 1.20	0.3987
Folate, DFE (µg)	3.82 ± 0.43	0.82 ± 0.52	<0.0001	12.3 ± 0.5	9.34 ± 1.57	0.3099
Vitamin B_6_ (mg)	2.18 ± 0.32	1.25 ± 0.51	0.1048	12.9 ± 0.6	10.9 ± 1.4	0.4282
Vitamin B_12_ (µg)	0.86 ± 0.15	0.11 ± 0.08	<0.0001	4.35 ± 0.33	0.76 ± 0.40	<0.0001
Vitamin C (mg)	19.2 ± 0.9	9.39 ± 1.87	0.0240	45.1 ± 0.8	34.8 ± 1.8	0.0138
Vitamin D (µg)	91.1 ± 0.5	86.4 ± 2.0	0.3103	95.4 ± 0.3	93.2 ± 1.5	0.3487
	% population above Adequate Intake (AI)					
Dietary fiber (g)	0.62 ± 0.08	2.03 ± 0.71	0.0158	5.81 ± 0.29	16.8 ± 1.2	<0.0001
Potassium (mg)	33.2 ± 0.8	53.1 ± 2.0	<0.0001	31.3 ± 0.6	57.3 ± 1.6	<0.0001
Sodium (mg)	99.9 ± 0.03	99.9 ± 0.1	0.1421	99.2 ± 0.1	99.2 ± 0.4	0.9242

Values are means ± standard error of means. *p* values are for difference between consumers and non-consumers. RAE, retinol activity equivalents; DFE, dietary folate equivalent.

**Table 4:** Healthy Eating Index (HEI)-2015 scores for children and adult yogurt consumers and non-consumers. (NHANES 2001–2016, gender combined data).

	Children (2–18 Years)				Adults (≥19 Years)			
	Non-Consumers (*n* = 22,335)	Consumers (*n* = 1557)	β	*p* Value for Difference	Non-Consumers (*n* = 34,711)	Consumers (*n* = 2024)	β	*p* Value for Difference
HEI-2015 total score	46.6 ± 0.2	51.4 ± 0.5	4.8 ± 0.5	<0.0001	50.2 ± 0.1	57.8 ± 0.4	7.6 ± 0.4	<0.0001
Component 1 (total vegetables)	2.16 ± 0.02	2.05 ± 0.05	−0.12 ± 0.06	0.0419	3.11 ± 0.01	3.10 ± 0.04	−0.01 ± 0.04	0.7977
Component 2 (greens and beans)	0.83 ± 0.02	1.02 ± 0.07	0.19 ± 0.07	0.0082	1.46 ± 0.02	1.79 ± 0.06	0.33 ± 0.06	<0.0001
Component 3 (total fruit)	2.45 ± 0.03	2.99 ± 0.08	0.54 ± 0.08	<0.0001	2.06 ± 0.02	2.88 ± 0.06	0.82 ± 0.06	<0.0001
Component 4 (whole fruit)	2.12 ± 0.03	2.85 ± 0.08	0.73 ± 0.08	<0.0001	2.01 ± 0.02	3.05 ± 0.06	1.04 ± 0.06	<0.0001
Component 5 (whole grains)	2.14 ± 0.04	2.55 ± 0.14	0.41 ± 0.14	0.0043	2.38 ± 0.03	3.29 ± 0.11	0.91 ± 0.11	<0.0001
Component 6 (dairy)	6.89 ± 0.04	8.09 ± 0.10	1.20 ± 0.10	<0.0001	4.95 ± 0.03	6.93 ± 0.07	1.98 ± 0.07	<0.0001
Component 7 (total protein foods)	3.55 ± 0.02	3.40 ± 0.06	−0.15 ± 0.06	0.0137	4.20 ± 0.01	4.20 ± 0.04	−0.00 ± 0.04	0.9635
Component 8 (seafood and plant protein)	1.53 ± 0.02	1.86 ± 0.10	0.33 ± 0.10	0.0009	2.25 ± 0.02	2.74 ± 0.07	0.49 ± 0.11	<0.0001
Component 9 (fatty acid ratio)	3.82 ± 0.04	3.51 ± 0.15	−0.31 ± 0.15	0.0442	5.02 ± 0.03	4.70 ± 0.11	−0.32 ± 0.11	0.0053
Component 10 (sodium)	4.91 ± 0.04	5.69 ± 0.13	0.78 ± 0.13	<0.0001	4.16 ± 0.03	4.91 ± 0.10	0.75 ± 0.11	<0.0001
Component 11 (refined grain)	5.20 ± 0.04	5.96 ± 0.13	0.76 ± 0.13	<0.0001	6.15 ± 0.03	7.03 ± 0.10	0.88 ± 0.10	<0.0001
Component 12 (saturated fat)	5.49 ± 0.04	5.92 ± 0.15	0.43 ± 0.16	0.0084	5.97 ± 0.03	6.46 ± 0.10	0.49 ± 0.10	<0.0001
Component 13 (added sugar)	5.52 ± 0.04	5.51 ± 0.11	−0.01 ± 0.11	0.9766	6.51 ± 0.04	6.78 ± 0.09	0.27 ± 0.09	0.0036

Values are least square means ± standard error of means, adjusted for age, gender, ethnicity, poverty income ratio, physical activity level, and current smoking status and alcohol (only for ≥19 years). *p* values are for difference between consumers and non-consumers. β is the difference between consumers and non-consumers.

**Table 5:** Association of yogurt consumption with anthropometric measures in children and adults. (NHANES 2001–2016, gender combined data).

Variables	Non-Consumer		Consumer		*p* Value for Difference
	*n*	LSM ± SEM	*n*	LSM ± SEM	
Children (2–18 years)					
BMI z-Score	21,949	0.49 ± 0.02	1520	0.43 ± 0.04	0.2140
Weight (kg)	22,100	43.0 ± 0.2	1536	42.2 ± 0.4	0.0311
Waist Circumference (cm)	21,615	68.9 ±0.2	1456	68.1 ± 0.4	0.0635
Adults (≥19 years)					
BMI (kg/m^2)^	34,133	28.8 ± 0.1	1995	27.9 ± 0.2	<0.0001
Weight (kg)	34,274	82.4 ± 0.2	1998	80.3 ± 0.6	0.0004
Waist Circumference (cm)	33,416	98.8 ± 0.2	1947	96.5 ± 0.5	<0.0001

Values are adjusted for age, gender, ethnicity, poverty income ratio, physical activity level, current smoking status and alcohol (only for 19 years and older), and kcal. *p* values are for difference between consumers and non-consumers. LSM, least square means; SEM, standard error of means; BMI, body mass index.

## Supplementary Materials

The following are available online at https://www.mdpi.com/2072-6643/12/11/3435/s1, Table S1: Demographics associated with yogurt consumption in children and adults (NHANES 2001-2016, gender combined data).
