# Supplementary material for: Association of Yogurt Consumption with Nutrient Intakes, Nutrient Adequacy, and Diet Quality in American Children and Adults
Source: Nutrients. 2020 Nov 9;12(11):3435. doi: 10.3390/nu12113435 (PMC7696083; doi:10.3390/nu12113435)
Supplement: Supplementary file 1 [file nutrients-12-03435-s001.pdf]

Supplemental Table 1. Demographics associated with yogurt consumption in children and adults (NHANES 2001-2016, gender combined data).

Variables	Children (2-18 years)			Adults (≥19 years)		
	Non-Consumers	Consumers	P Value for difference	Non-Consumers	Consumers	P Value for difference
Age (years)	10.3 ± 0.1	7.26 ± 0.16	<0.0001	46.7 ± 0.2	49.3 ± 0.5	<0.0001
Gender (% Male)	50.7 ± 0.5	50.4 ± 1.7	0.8391	50.3 ± 0.3	36.3 ± 1.5	<0.0001
Ethnicity						
Mexican American (%)	14.2 ± 1.0	12.5 ± 1.2	0.1391	8.46 ± 0.64	5.09 ± 0.56	<0.0001
Non-Hispanic White (%)	56.6 ± 1.6	66.4 ± 1.7	<0.0001	68.2 ± 1.2	79.9 ± 1.3	<0.0001
Non-Hispanic Black (%)	15.0 ± 0.9	6.36 ± 0.71	<0.0001	11.9 ± 0.7	4.29 ± 0.39	<0.0001
Poverty Income Ratio						
< 1.35 (%)	34.9 ± 1.0	24.6 ± 1.8	<0.0001	23.8 ± 0.7	14.5 ± 1.1	<0.0001
1.35 - 1.85 (%)	11.0 ± 0.4	8.36 ± 1.02	<0.0001	9.89 ± 0.26	7.72 ± 0.86	0.0116
> 1.85 (%)	54.1 ± 1.1	67.0 ± 2.0	<0.0001	66.3 ± 0.8	77.8 ± 1.4	<0.0001
Physical Activity						
Sedentary (%)	12.4 ± 0.4	11.9 ± 1.5	0.7302	26.6 ± 0.5	18.6 ± 1.1	<0.0001
Moderate (%)	20.8 ± 0.5	17.0 ± 1.5	0.0176	35.1 ± 0.4	36.5 ± 1.3	0.2862
Vigorous (%)	66.7 ± 0.6	71.1 ± 1.9	0.0251	38.3 ± 0.6	44.9 ± 1.6	<0.0001

Values are means ± standard error of means. P values are for difference between consumers and non-consumers.
